# Tongue Traction as a Respiratory Stimulant in Asphyxia, with Report of Cases

**Published:** 1893-11

**Authors:** T. W. Shearer

**Affiliations:** Wallisville, Texas


For Texas Medical Journal.

### TONGUE TRACTION AS A RESPIRATORY STIMULANT IN ASPHYXIA, WITH A REPORT OF THREE CASES.

BY T. W. SHEARER, M. D., WALLISVILLE, TEXAS.

THE MOST valuable fact that has appeared in print during the last year is Laborde's method of "Procedure of the Tongue," which consists in placing the patient in as favorable a position as possible,—depending upon nature and cause of asphyxia,—and then grasping the tongue with a pair of forceps, or placing the finger at the root of the tongue, and rythmically withdrawing the tongue and allowing it to retract ten to thirty times a minute, according to age and condition of patient.

By this method we have saved the lives of three children who otherwise must have died.

CASE I. This was a labor of considerable length, in which chloroform was used, and finally had recourse to forceps. The head of the child was considerably injured by forceps, one blade engaging the head about the left eye, and the other the occiput on the right side. The child was apparently lifeless. The head was immediately moulded into shape with the hands. There was considerable depression at the point of contact of the blades; this was corrected by making pressure in different directions with the palmar surface of the hands. Artificial respiration was then carefully and earnestly indulged in for some time, with no apparent result. The tongue was then grasped with a pair of common dressing forceps. It is not necessary to lacerate the tongue with a tenaculum, as forceps or the fingers serve every purpose. By means of the forceps, the tongue was withdrawn and allowed to retract in a rythmical manner, and in a few minutes the child gasped, and was soon breathing in a natural manner.

CASE 2. A primipara, who had been treated for gonorrhea two years previous to confinement, had a long protracted labor due to inertia of the uterus, caused by gelatinous degeneration of the amniotic fluid which rendered its escape impossible until after the birth of the child. Chloroform was used for several hours during the latter part of the labor. The child was very much asphyxiated. The respiratory tract seemed full of gelatinized fluid. After compressing the chest and removing as much of the offending material as possible, tongue traction was applied for about fifteen minutes, and I was rewarded with success.

CASE 3 is very interesting. Mother, a multipara. An emergency case. Was called while passing the house; found the woman in last stage of labor, with breech presentation. The child was born very much asphyxiated. Considerable difficulty was experienced in performing tongue traction with the finger, so a messenger was dispatched for pocket case; and in the meantime artificial respiration was applied faithfully, with no result. On arrival of the forceps, they were applied to the tongue, and traction commenced rythmically for some minutes. Success seemed almost hopeless, when the thought occurred that possibly the conjoined method of artificial respiration and tongue traction might succeed. Accordingly, while performing traction with the right hand, the left hand was placed upon the chest, and as the tongue was withdrawn from the mouth the chest was compressed, relaxing quickly as the tongue retracted. This was rewarded with success in less than one minute, and it must certainly be regarded as a valuable aid to the Laborde's method.

